# Preparation of Nanocomposite Biopolymer Films from *Commelina coelestis* Willd Starch and Their Nanostructures as a Potential Replacement for Single-Use Polymers

**DOI:** 10.3390/foods13244129

**Published:** 2024-12-20

**Authors:** Lucia García-Guzmán, Gonzalo Velazquez, Israel Arzate-Vázquez, Patricia Castaño-Rivera, Maria Guerra-Valle, Johanna Castaño, Andrea Y. Guadarrama-Lezama

**Affiliations:** 1 Facultad de Química, Universidad Autónoma del Estado de México, Paseo Colón esq. Paseo Tollocan s/n, Col. Residencial Colón, Toluca 50120, Estado de Mexico, Mexico; lucia.gg@sfelipeprogreso.tecnm.mx; 2 Tecnológico Nacional de México/Tecnológico de Estudios Superiores de San Felipe del Progreso, División Ingenieria Civil, Avenida Instituto Tecnológico S/N, Ejido, Tecnológico, San Felipe del Progreso 50640, Estado de México, Mexico; 3 Instituto Politécnico Nacional, CICATA, Unidad Querétaro, Cerro Blanco 141, Colinas del Cimatario, CP, Santiago de Querétaro 76090, Querétaro, Mexico; gvelazquezd@ipn.mx; 4 Centro de Nanociencias y Micro y Nanotecnologías, Instituto Politécnico Nacional, Luis Enrique Erro s/n, Zacatenco, Gustavo A. Madero, Ciudad de México 07738, Mexico; iarzate@ipn.mx; 5 Unidad de Desarrollo Tecnológico UDT, Universidad de Concepción, Coronel 4190000, Chile; p.castano@udt.cl; 6 Escuela de Nutrición y Dietética, Facultad de Ciencias para el Cuidado de la Salud, Campus Concepción, Universidad San Sebastián, Lientur 1457, Concepción 4080871, Chile; maria.guerra@uss.cl; 7 Department of Chemical Engineering, Universidad de Concepción, Concepción P.O. Box 160-C, Chile

**Keywords:** CNCs: cellulose nanocrystals, CNFs: cellulose nanofibers, SNCs: starch nanocrystals, reinforcement, dimensional stability, nanocomposite biopolymer films

## Abstract

This study explored the effect of incorporating cellulose and starch nanoparticles, obtained from the *Commelina coelestis* Willd plant, on the physical and chemical properties of starch-based films derived from the same plant. Additionally, the synergistic effect of combining the nanostructures was assessed. The nanocomposite biopolymer films were prepared by the casting method using 1 and 3 wt% concentrations of the nanostructures (CNCs: cellulose nanocrystals, CNFs: cellulose nanofibers, SNCs: starch nanocrystals), or their blend. The physicochemical (swelling capacity and water solubility), morphological (SEM and AFM), thermal (DSC and TGA), and mechanical properties (tensile strength, elongation at break, and Young’s modulus) of the films were evaluated. The nanocomposite biopolymer films exhibited better dimensional stability (40–60%) than the control films. Tensile strength (8–300%) and Young’s modulus (15–690%) were improved. Moreover, these films displayed enhanced thermal stability, withstanding temperatures exceeding 305 °C. FTIR spectra evidenced intermolecular interaction among the matrix and nanostructures. Microscopic analyses further supported the integrity of the films, which displayed a homogeneous surface and the absence of fractures. In addition, the nanocomposite biopolymer films prepared with 1 wt% cellulose nanocrystals and nanofibers had a lower opacity than those with a higher percentage (3 wt%). Overall, our findings suggest that the *Commelina coelestis* Willd is a promising starch source that can be used to obtain nanocomposite biopolymer films as an alternative to produce novel, efficient, and eco-friendly materials with adequate thermo-mechanical properties intended to replace conventional plastic materials in single-use applications such as those used in the food packaging industry.

## 1. Introduction

The extensive use of plastics in recent decades has notably increased the environmental problems derived from the large amount of waste generated [1,2]. The packaging industry is the primary user of plastics worldwide, accounting for 39.6% of total usage [3]. Developing novel environmentally sustainable materials for food packaging is a challenge to address to offer new alternatives for waste reduction.

Polysaccharides are widely used to prepare films, mainly due to their abundance and low toxicity [4]. Among the polysaccharides, starch and cellulose are excellent choices to produce biodegradable films intended for food packaging due to their high availability and low cost [2,5]. However, the mechanical performance and gas barrier capacity of these films depend significantly on the use of additives [6]. Several starch and cellulose sources including corn [7,8], rice [9,10], wheat [11], potato [12], pea [13], amaranth [14], and banana [15] have been used as raw materials to prepare films. The final characteristics of the films depend on the botanical source and processing conditions of the polysaccharide [4]. Although the starch and cellulose isolated from many sources have yet to be studied, to the best of the author’s knowledge, there have been no studies in which starch or cellulose as well as nanostructures were isolated from the *Commelina coelestis* Willd (CCW) plant root to prepare additive films with these nanostructures. This native plant of Mexico is a species of the *Commelina* genus, widely found throughout the country, and is traditionally used as a medicinal plant [16,17]. Although its root can be cooked and consumed, most of the time it is considered as agro-waste, making the final disposal an environmental problem. Isolating and characterizing starch and cellulose from this plant could help to find alternative uses for this natural resource.

A constant challenge in the development of starch-based biodegradable films is to improve their mechanical properties [18]. Many studies have focused on the preparation of micro- or nanoscale structures such as nanofibers, cellulose nanocrystals, or starch nanocrystals as reinforcing materials to be added to starch-based films to improve their mechanical and barrier properties [1,19,20,21]. Cellulose nanocrystals (CNCs) are needle-like structures with dimensions of 10–20 nm in width and diameter between 1 and 50 nm and are typically obtained by acid hydrolysis. In contrast, cellulose nanofibers (CNFs) are fibrillar structures resulting from the linear combination of cellulose chains in amorphous and crystalline forms, conforming a long network matrix [22,23]. Starch nanocrystals (SNCs) are platelet-shaped crystalline structures formed by the double helices of amylopectin chains, with dimensions of 20–40 nm in length, 15–30 nm in width, and a thickness of 5.7 nm [24,25].

All three types of nanoparticles, namely CNCs, CNFs, and SNCs, are distinguished by their unique structures, low density, high stiffness, high elastic modulus, and high surface area [26]. These properties, coupled with the presence of various functional groups, result in adaptable functionalities for a wide variety of applications in various biotechnological and industrial sectors [27,28,29]. Moreover, it is possible to modify the surfaces of nanostructures to improve compatibility with other polymers as nano-reinforcements due to the abundant presence of hydroxyl groups [3,30]. Nanostructures (nanocrystals and nanofibers) have been prepared from the starch and cellulose isolated from CCW, obtaining good chemical, mechanical, and thermal properties [31].

In recent years, research has focused on the development of bionanocomposites with improved functional, thermal, and mechanical properties. Clear examples of this technology are nanocomposite biopolymer films based on starch as a continuous phase and an organic or inorganic filler of nanometric size as a discontinuous phase (dispersed) in the film matrix. These fillers or reinforcements reduce the mobility of the polymer chains and, in some cases, reduce the water vapor permeability and swelling capacity of the nanocomposite biopolymer films, allowing for superior properties without affecting the density, transparency, and processability [32,33].

This study aimed to evaluate the effect of the type of nanostructure (CNFs, CNCs, and SNCs) and nanostructure content on the physicochemical, thermal, and mechanical properties of *Commelina coelestis* Willd nanocomposite biopolymer starch-based films as a sustainable alternative to prepare non-biodegradable materials intended for food packaging.

## 2. Materials and Methods

### 2.1. Materials

Starch was extracted from the root of *Commelina coelestis* Willd. The root was collected in the northwest zone of the city of Atlacomulco, State of Mexico (coordinates 19°50′12.8″ N 99°54′52.2″ W). The isolated *Commelina* starch (SC) contained 26% amylose and 74% amylopectin. Glycerol (>99% purity) was purchased from Sigma-Aldrich Ltd. All reagents used were of analytical grade.

The isolation of nanostructures, cellulose nanofibers (CNFs), cellulose nanocrystals (CNCs), and starch nanocrystals (SNCs) from *Commelina* root followed the procedure reported elsewhere [31]. Characterization revealed average sizes of 34 nm for the CNFs, 43–44 nm for the CNCs, and 22–27 nm for the SNCs.

### 2.2. Nanocomposite Biopolymer Film Preparation

**Figure 1:**
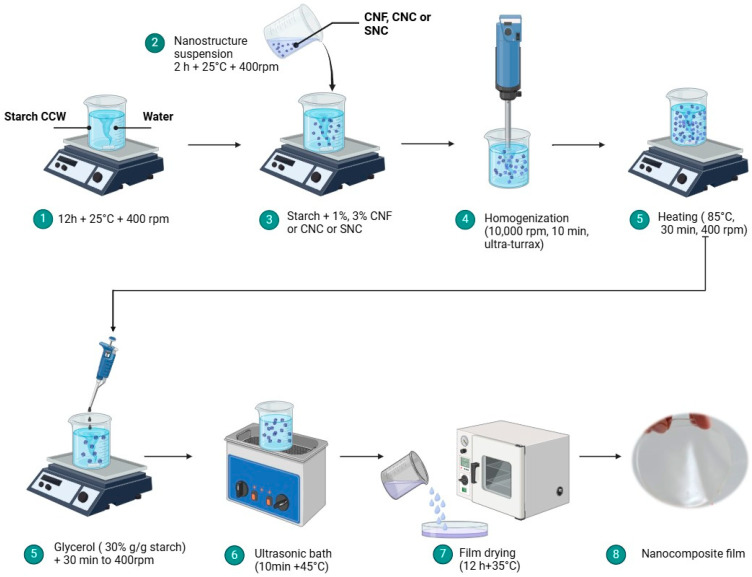
Preparation process of the nanocomposite biopolymer films.

Eleven formulations of nanocomposite biopolymer films based on *Commelina coelestis* Willd (CCW) starch were prepared following the methodology of Coelho et al. [34] and Castaño et al. [35]. A solution of starch was prepared by dissolving 2.5 g of starch in 100 mL of distilled water. Subsequently, 1% or 3% of CNFs, CNCs, SNCs, and the combination of CNC-CNF and SNC-CNF, previously hydrated for 2 h at 25 °C and 400 rpm, were added to the solution based on the dry weight of starch. The solutions were homogenized for 10 min at 10,000 rpm using an Ultra-Turrax disperser (Tissue Tearor, 985370, Biospec Products, Colton, CA, USA). Subsequently, the homogenized solutions were heated at 85 °C for 30 min under constant agitation (400 rpm). Following this, glycerol (30% dry starch base) was incorporated as a plasticizing agent into the solutions and mixed for 30 min. The resulting filmogenic solution was ultrasonicated in an ultrasonic bath (model M2800H, Branson, San Diego, CA, USA) at 45 °C for 10 min. The filmogenic solution was dispersed in glass Petri dishes (90 × 15 mm) and dried at 35 ± 1 °C for 12 h in a forced convection oven (Riossa, RSU Labsupply, Monterrey, Mexico). Subsequently, the nanocomposite biopolymer films were stored at room temperature in plastic bags inside a desiccator with silica gel. The codes used in this study were F1 for the starch-based film, and CNF_1_ and CNF_3_ for the nanocomposite biopolymer films reinforced with nanofibers of cellulose (CNF) at 1% and 3%, respectively. CNC_1_ and CNC_3_ were added with nanocrystals of cellulose (CNCs) at 1% and 3%, respectively, and SNC_1_ and SNC_3_ were added with nanocrystals of starch (SNCs) at 1% and 3%, respectively. The nanocomposite biopolymer films with the combination of CNC-CNF and SNC-CNF were labeled as CNC_1_-CNF_1_, CNC_3_-CNF_3_, SNC_1_-CNF_1_, and SNC_3_-CNF_3_, respectively. The preparation process of the nanocomposite biopolymer films is shown in Figure 1.

### 2.3. Characterization of the Nanocomposite Biopolymer Films

#### 2.3.1. Swelling Capacity and Water Solubility

The swelling capacity and water solubility of the films were measured according to the methodology reported by Zhang et al. [36]. Discs of the films (2 cm in diameter) were cut and weighed (m_0_), after which they were dried for 24 h at 105 °C in an oven and weighed again (m_1_). The dried films were immersed in 50 mL of distilled water in a 100 mL beaker at 25 °C for 24 h. The film pieces were then removed and dried for 24 h at 105 °C, and the final dry mass (m_2_) was recorded. Swelling capacity and solubility were calculated using Equations (1) and (2), respectively.

Swelling capacity (%) = 100 × ((m_0_ − m_1_))/m_0_ (1)

Solubility (%) = 100 × ((m_1_ − m_2_)/m_1_) (2)

#### 2.3.2. Opacity

The opacity of the films was determined according to the method reported by Ren et al. [37]. The films were cut into rectangular pieces (1 × 4 cm) and placed directly into the test cell of a UV spectrophotometer (PerkinElmer UV–Vis spectrometer, Lambda 25, Shelton, CT, USA), and the absorbance was measured at 600 nm. The empty test cell was used as a reference. Opacity was calculated using the equation:

(3) Opacity (O)=Abs600d

where O is the opacity, *Abs*_600_ is the value of absorbance at 600 nm, and *d* is the film thickness (mm). A higher value of O indicates a lower degree of transparency [38].

#### 2.3.3. Scanning Electron Microscopy (SEM)

Micrographs of the surface and cross-section of the films were taken using scanning electron microscopy (Jeol JSM-IT100, Tokyo, Japan). The samples were mounted on an aluminum sample holder with carbon-coated double-sided adhesive tape, after which they were coated with gold and examined under an accelerating voltage of 5 kV. Images at different magnifications were taken on the surface and cross-section. For the examination of the cross-section of the films, the samples were fractured after freezing them with liquid nitrogen.

#### 2.3.4. Atomic Force Microscopy (AFM) Analysis

Topographic images were obtained using an AFM instrument (MultiMode V connected to a NanoScope V minicontroller, Bruker, Billerica, MA, USA). Sample fragments were attached to the sample holders with double-sided adhesive tape. The images were obtained in the air using RTESP probes (Bruker, Billerica, MA, USA). Topographic images of different scanning areas were captured: 10 × 10 µm^2^, 5 × 5 µm^2^, and 1 × 1 µm^2^. Roughness parameters (Ra and Rq) were measured on the highest resolution images (1 × 1 µm^2^) using NanoScope v. 1.40 software (Bruker). Tapping mode was the preferred method in this work, as it is the most widely used in food science.

#### 2.3.5. Differential Scanning Calorimetry (DSC)

The samples were analyzed using a calorimeter (STARE System, Mettler Toledo Inc., Zürich, Switzerland). A 3 mg sample was placed in a 40 μL aluminum standard pan, and 7 μL of distilled water was added. The pan was then sealed and heated from 30 to 135 °C at 10 °C/min in the calorimeter. An empty pan was used as the reference. The software STAR SW version 9.30 was used for data analysis.

#### 2.3.6. Thermogravimetric Analysis (TGA)

Thermal stability of the samples was evaluated using a thermogravimetric analyzer (TGA Q500, TA Instruments, New Castle, DE, USA) according to the procedure reported by Hernández-Hernández et al. (2015). Each sample (about 2 mg) of film was heated from 25 to 600 °C and then from 600 to 700 °C at a rate of 10 °C/min in an atmosphere of nitrogen and oxygen, respectively.

#### 2.3.7. Fourier Transform Infrared Spectroscopy (FTIR)

FTIR analysis was carried out using an IR spectrophotometer (Spectrum Two, PerkinElmer Inc., Hopkinton, MA, USA) with an ATR accessory. Spectra were recorded in the range from 4000 to 400 cm^−1^ by accumulating 16 scans at a resolution of 4 cm^−1^.

#### 2.3.8. Mechanical Properties

Tensile strength (TS), elongation at break (EB), and Young’s modulus (Y) were measured using a texture instrument (Lloyd Instruments Model TAPlus). Rectangular film samples (10 × 100 mm) were cut and preconditioned at 57% RH for 24 h before measuring. The initial grip separation was 40 mm, and the crosshead speed was set at 1 mm s^−1^. At least three specimens from each film were tested at room temperature.

### 2.4. Statistical Analysis

All measurements were made in triplicate, and the quantitative data were presented as the mean ± standard deviation. Statistical analysis was carried out by one-way analysis of variance (one-way ANOVA) using the Minitab 22.1 software (USA), considering significant differences when *p* < 0.05. In addition, a Tukey test was assessed.

## 3. Results and Discussion

### 3.1. Swelling Capacity and Solubility of the Nanocomposite Biopolymer Films

Swelling capacity (SC) and water solubility (WS) are important parameters for assessing the quality and applicability of nanocomposite biopolymer films, particularly for food packaging.

The introduction of a nanostructure into the starch-based film resulted in a considerable reduction in SC (38–62%) compared with the pure starch film (Table 1). This reduction could be attributed to the interaction between CNF/CNC/SNC and starch, which decreased the water adsorption [38,39]. The results for SC were according to those reported for yam and high amylose maize starch but lower than those reported for B-type, thus waxy potato starch (50–80%), sweet potato starch (17–20%), and potato starch (32–36%) [40].

In developing starch-based food packaging, it is important to understand the factors affecting starch granule swelling and to explore the relationship between starch granule swelling and sticking, gelling, cooking, product quality, and nutrition [40]. For example, Bangar et al. [41] noted that the higher the swelling power of native starch granules, the softer and easier to disintegrate the materials that are produced [41].

Improved dimensional stability of the films was observed with the incorporation of the nanostructures. According to El Halal et al. [42], the addition of cellulose fibers to starch films increases the interactions between the starch molecules and hydroxyl groups of cellulose, resulting in a decreased interaction with water molecules. Similar results were observed studying the interaction between the carbohydrate matrix of films based in thermo-plasticized starch and cellulose fibers [43]. The crystallinity of the nanostructures acts as a barrier, limiting the swelling of the polymer matrix molecules and reducing the water sorption [44]. A decrease in swelling capacity and water solubility favor the development of materials for food packaging applications [45,46]. The SC of the films with the added combination of cellulose nanostructures (CNC_3_-CNF_3_) had a significant difference (*p* ≤ 0.05), and the highest value was observed at the higher concentration (3%). Conversely, the swelling capacity of films containing starch nanocrystals and cellulose nanofibers (SNC-CNF) showed no significant differences. This behavior was related to the hydrophobic character of the cellulose fibers in comparison to the starch hydrophilic property [43].

Water solubility indicates the ability of films to withstand exposition to water. Low solubility is desirable to avoid the disintegration of the film under a high-humidity environment [47]. The incorporation of cellulose nanocrystals and nanofibers resulted in a notable reduction in the water solubility of the films, with values ranging from 23.3% to 23.9% in comparison to the pure starch-based film (Table 1). This decline in solubility can be attributed to the reduced affinity of nanocellulose for water in comparison to starch [36]. This behavior was observed in the CNC corn starch films by Slavutsky et al. [19] and in the CNC cassava starch films with bamboo bagasse CNCs by Thipchai et al. [48], where a higher concentration of cellulose nanostructures resulted in a decrease in the water solubility of the films.

The addition of starch nanocrystals resulted in a slight reduction in solubility when compared to the pure starch film, except for the SNC_3_ sample. The capacity of starch nanocrystals (SNCs) to reduce the solubility of the films is contingent upon the hydrogen bonds between the hydroxyl groups of the SNC and the starch matrix. These bonds facilitate the formation of a compact three-dimensional (3D) network structure, which limits the interaction of the films with water [49,50]. Nanocomposite biopolymer films with higher SNC concentrations (SNC_3_) exhibited higher solubility, which can be attributed to the possibility that some of the SNCs may not have been integrated into the 3D network of the films, thereby becoming accessible for interaction with water.

The nanocomposite biopolymer film comprising a mixture of cellulose nanostructures with a concentration of 3% (CNC_3_-CNF_3_) exhibited a notable reduction in solubility, with a decrease of approximately 24% compared to the control film. In contrast, the films containing only 1% of nanostructures (CNC_1_-CNF_1_, SNC_1_-CNF_1_) demonstrated a relatively unchanged solubility, with no statistically significant difference (*p* ≤ 0.05) observed. This indicates that at concentrations below 3%, the solubility remains statistically equivalent to that of the film comprising pure starch F1.

The property of opacity plays a fundamental role in the development of nanocomposite biopolymer films for use in the food packaging industry. This particular property represents a fundamental aspect that serves to define the quality of the nanocomposite films, functioning as a critical factor in their overall performance and potential applications [44,51,52]. Since film thickness is a factor directly related to opacity [53], it was strictly controlled in this study to avoid uncertainty in the comparisons. The opacity values are shown in Table 1. Relatively low opacity values were obtained in the films with 1% nanostructure. In samples containing 3% of nanostructures, the opacity increased slightly, indicating that the transparency of the starch films decreased when the CNF, CNC, or SNC content increased. The SNC_3_ films exhibited the highest opacity index, which was attributed to the distribution of starch nanocrystals in the interspaces of the films, hindering light transmission. Particle sizes below 40–50 nm have been reported as adequate to maintain the transmitted light intensity [24]. In general, these opacity results can be explained by (a) the increase in the density of the composite films due to strong interactions between the CNF, CNC, and SNC nanostructures and the starch matrix, and (b) changes in the light scattering caused by the incorporation of nanostructures [53,54,55].

### 3.2. Microstructure Analysis of the Nanocomposite Biopolymer Films

**Figure 2:**
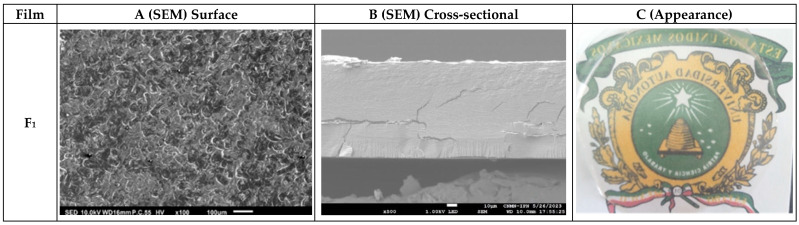

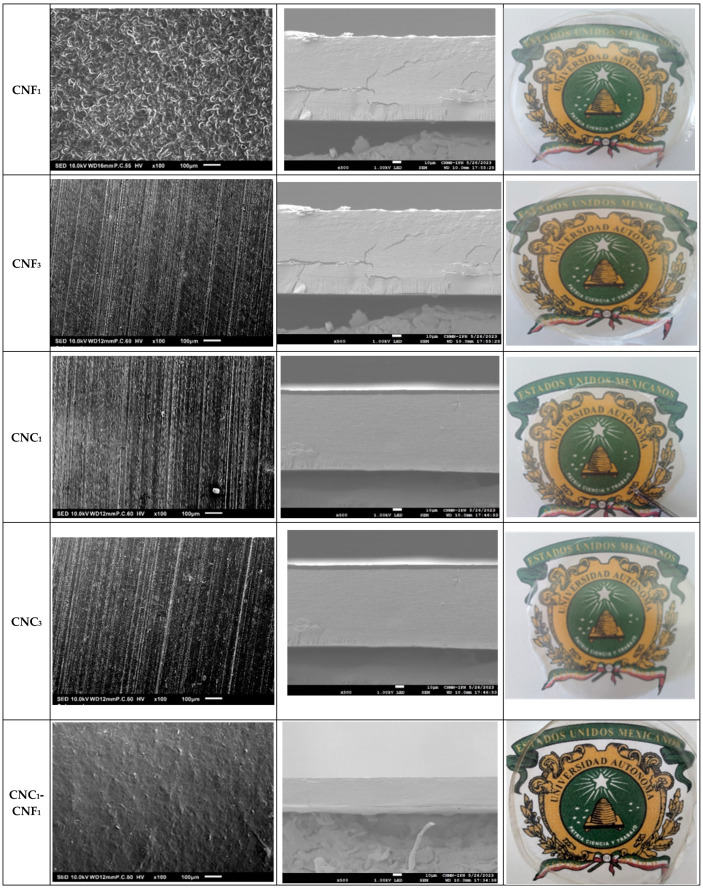

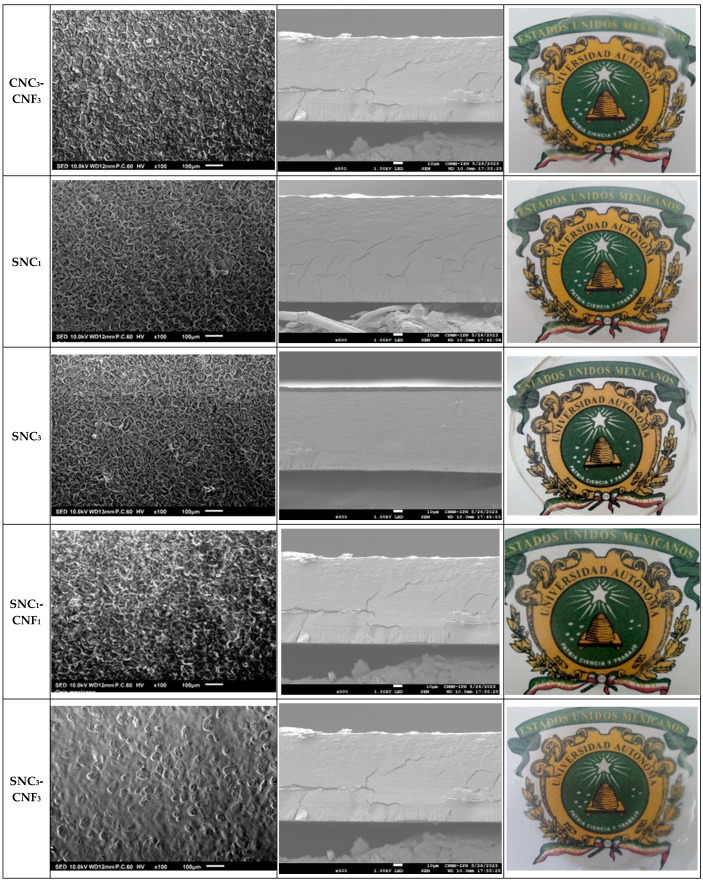
(**A**) Surface and (**B**) cross-sectional SEM micrographs. (**C**) Digital graphics of the CCW nanocomposite biopolymer films with different concentrations of CNFs, CNCs, and SNCs.

Homogeneity and compatibility are critical parameters of composite materials, indicating an effective dispersion of the components [56]. Figure 2 illustrates the surface (A) and cross-sectional (B) SEM micrographs of the starch nanocomposite films. All nanocomposite biopolymer films were homogeneous, translucent, and had a similar visual appearance to the pure starch film with a surface free of bubbles, with no pores, indicating good compatibility between the nanostructures and the starch matrix [57]. Pure starch films (F_1_) showed a continuous and homogeneously distributed surface with no pores or bubbles; with some structures known as withered granular envelopes (ghost). Similar structures have been reported in potato starch films [58], resulting from the structural collapse of starch granules during gelatinization. These ghosts exhibit depressions (dark region) that reflect the surface tension developed by the amylose solution in relation to the remaining starch granules as the liquid evaporates during the film drying process [59,60].

Figure 2B shows cross-sectional micrographs of the films. In F_1_(B), the starch film showed a continuous and homogeneous phase. In F1(A), there was no evidence of phase separation or starch granules within the film or on the surface, indicating that most starch granules gelatinized during film formation due to the plasticizing effect of glycerol [36]. The surface of the nanocomposite biopolymer films with CNFs and CNCs (Figure 2B) showed a homogeneous and continuous structure. However, a smooth cross-sectional surface with slight beach marks [61] was observed. There were no pores in the fractured films, indicating the good compatibility between CNFs, CNCs, and the starch matrix [54,62]. These beach marks have been found in nanocomposite biopolymer films, indicating that nanostructures such as nanofibers and cellulose nanocrystals prevent crack propagation and produce twisted cracks through the weakest parts of the starch matrix [36,61]. This was more evident in films containing 3% of CNFs or CNCs. The CNF1 film showed a slight fracture at the top, probably due to sample processing, while the CNC_3_-CNF_3_ film showed a crack in the middle part, which crossed from end to end, probably because of the aggregation of the added nanostructures.

The nanocomposite biopolymer films prepared with 3% of CNC and SNC nanostructures showed a denser surface, with no apparent fractures and some agglomerates in the cross-sectional cut, compared to those with only 1% of nanostructures. Similar results have been reported for potato starch films with turmeric cellulose nanofibers [63]. The SNC_3_ nanocomposite biopolymer films showed the surface with more uniformly distributed beach marks, unlike its counterpart CNC_3_-CNF_3_, showing that aggregation of the nanostructures could occur at the same concentration. Adding CNFs at 3% or a combination of nanostructures in the CNC_3_-CNF_3_, SNC_1_-CNF_1_, and SNC_3_-CNF_3_ films resulted in slight fractures. On the other hand, the addition of nanofibers and nanocrystals of cellulose in combination (CNC_1_-CNF_1_) did not show fractures in the cross-sectional cut of the films. This may be attributed to a better interaction of nanostructures among them and to the polysaccharide nature of the film.

**Figure 3:**
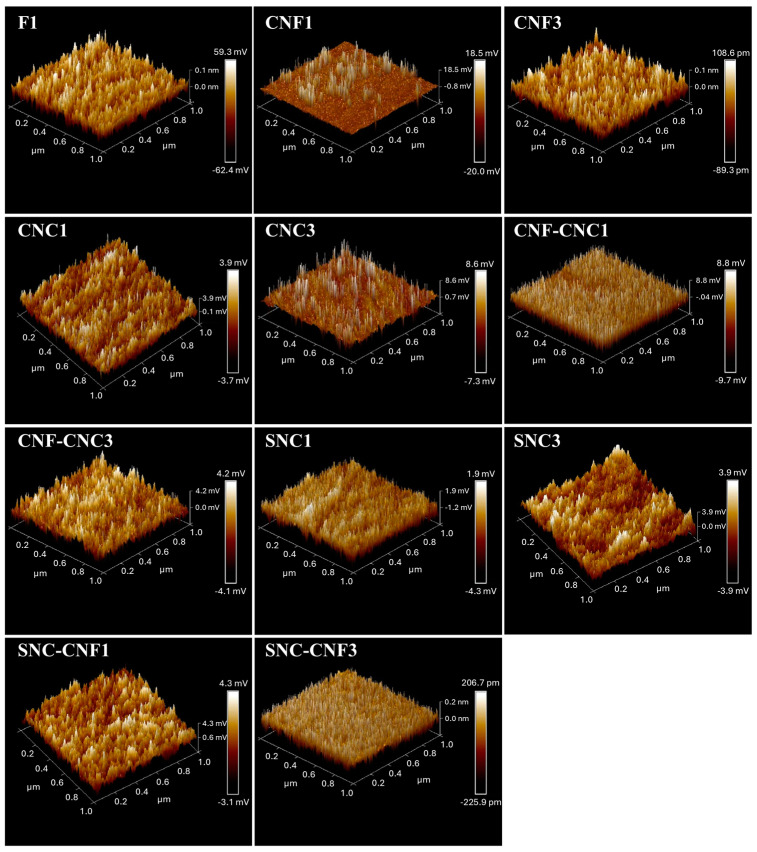
AFM-3D images of the CCW starch (F1) and nanocomposite biopolymer films (with 1 and 3%).

AFM analysis was carried out to determine the supramolecular structure of the nanocomposites. The AFM topographic images of the starch and nanocomposite biopolymer films are shown in Figure 3. As seen in the figure, the surface texture of the films was modified with the addition of the nanostructures in the formulation. The homogeneous distribution of the nanostructures without agglomerations was consistent with the microstructure observed in the SEM images. The roughness values of the films obtained from the topography images through the root mean square roughness (Rq) and arithmetic mean roughness (Ra) are shown in Table 1. The films with added 1% cellulose nanostructures (nanofibers and nanocrystals) exhibited lower roughness parameter values compared to the control film. In contrast, it was observed that the film with 1% starch nanocrystals (SNC_1_) presented higher values of both roughness parameters than the control film. When the concentration of the three types of nanostructures was increased from 1 to 3%, a considerable increase in the values of the roughness parameters was observed. For the case of films containing a mixture of cellulose-based nanostructures (CNC_1_-CNF_1_, CNC_3_-CNF_3_), it was observed that the values of the roughness parameters were higher than the control film.

Finally, SNC_1_-CNF_1_ and SNC_3_-CNF_3_ films showed very similar roughness values compared to the pure starch film. The AFM results showed that the addition of nanostructures to the polymeric matrix (starch) strongly affected the roughness of the films. Some studies published in the literature have shown that the addition of nanostructures increases the roughness of polymeric films. Antoniou et al. [64] observed a significant increase in the Ra and Rq values when chitosan nanoparticles were added to tara gum edible films. Similar behavior was also reported by Azeredo et al. [65] in the case of chitosan films containing cellulose nanofibers. Therefore, it is possible that by adding nanostructures, we can modify in a controlled way the roughness of the polymeric films and consequently affect some other surface characteristics such as gloss and contact angle.

### 3.3. Thermal Analysis of Nanocomposite Biopolymer Films

Thermal analysis provides more accurate information about the effect of nanostructures on the thermal degradation and stabilization of nanocomposite materials (films) as well as their potential applications [66]. Table 2 summarizes the values of T_max_ (°C) and degradation (%) at T_max_ obtained by the TGA of the nanocomposite biopolymer films as well as the values of the glass transition temperature (T_g_), maximum temperature (T_max_), final temperature (T_f_), and enthalpy (ΔH) obtained by DSC.

**Figure 4:**
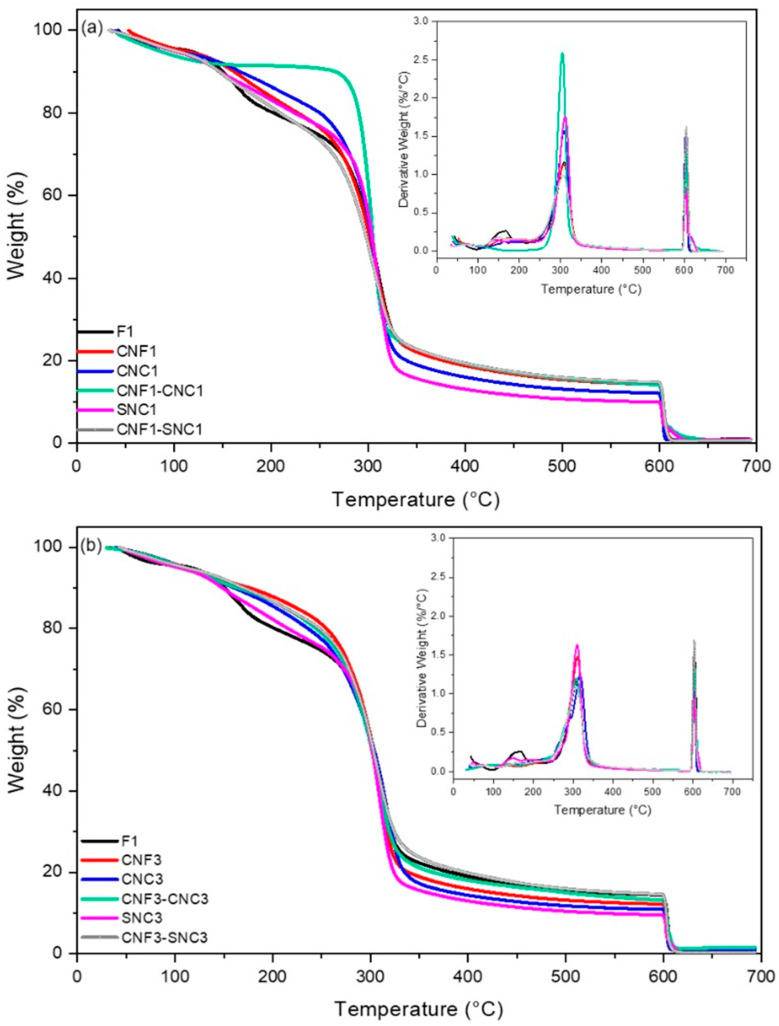
TGA and derivative curves of the nanocomposite biopolymer films with added (**a**) 1% and (**b**) 3% of nanostructures.

The TGA and derivative curves of F1 and the nanocomposite biopolymer films as a function of CNF, CNC, and SNC nanostructure content are shown in Figure 4. The nanocomposite biopolymer films exhibited improved thermal stability than those prepared only with starch, which can be associated with the effect of the nanostructures in hindering the “out-diffusion” of volatile molecules (e.g., glycerol) as well as the diffusion of oxygen into the polymer matrix. Both factors delay the decomposition process-based starch materials [67].

The thermal decomposition of all films took place in three stages; however, the type and concentration of nanoparticles influenced the thermal stability of the starch films.

The first phase of weight loss occurred between 60° and 150 °C, mainly due to the evaporation of water after the breaking of inter- and intra-molecular hydrogen bonds and the loss of volatile compounds [68]. Dehydration was more pronounced in the starch film (F_1_) compared to the nanocomposite biopolymer films containing nanostructures, especially the film with added nanofibers (CNF_1_ and CNF_3_), possibly due to the interaction of water with cellulose nanofibers [61].

The second and main stage of thermal decomposition occurred from 160 to 350 °C, which can be attributed to the decomposition of some low molecular weight polymers in the film matrix, the volatilization of glycerol, and the degradation of starch and nanostructures [69]. In this stage, the highest rate of thermal degradation occurred at 303 °C and 316 °C for the F1 and CNC_3_ nanocomposite biopolymer films, respectively. It is noteworthy that all of the nanocomposite biopolymer films showed a higher decomposition temperature compared to F1. In addition, the nanocomposite biopolymer films prepared with CNC_3_, followed by SNC_3_ and CNF at 3%, exhibited the highest degradation temperature (Table 2). The initial rate of weight loss was slower in the composites (Figure 4). In the second step, 5% of weight loss occurred at 167 °C for F1, while the composites with 1% and 3% of nanostructures were 174, 185, 166, 168, 170, 167, 168, and 171 °C for CNF_1_, CNF_3_, CNC_1_, CNC_3_, SNC_1_, SNC_3_, SNC_1_-CNF_1_, and SNC_3_-CNF_3_, respectively. The incorporation of nanostructures improved the thermal stability of the nanocomposite biopolymer films since the nanostructure hinders the “out-diffusion” of the volatile molecules (e.g., glycerol) as well as the diffusion of oxygen into the polymer matrix, retarding the decomposition process of the nanocomposite biopolymer films. Similar results were reported by Castaño et al., [67]. The removal of hydroxyl groups and the depolymerization and decomposition of carbon chains takes place in the films, contributing to the weight loss [70,71].

The final stage above 450 °C can be associated with inorganic impurities and residual carbon of the nanostructures and the starch-rich phase of the nanocomposite biopolymer films. The ash content of starch could be related to the presence of phosphate groups as well as calcium and magnesium [67,72].

All samples showed a similar trend in degradation, however, F1 presented the lowest values from 150 to 300 °C, while the film containing a blend of cellulose nanofibers and cellulose nanocrystals (CNC_1_-CNF_1_) presented the highest value. This shows (a) that the addition of nanostructures allows a more thermoresistant material to be obtained, and (b) the combination of nanostructures increases the resistance of the nanocomposite biopolymer films to high temperature. In terms of applicability as packaging materials, the addition of CNC_1_-CNF_1_ may allow for the design of a heat-sealable material that will not deform/degrade until reaching 300 °C, increasing the options of applicability in the food packaging industry.

Thermal properties obtained from the DSC thermograms of the films are also presented in Table 2. The DSC curves of the nanocomposite biopolymer showed a broad endothermic peak at around 205 °C, corresponding to the decomposition temperature. This transition is concomitant with the mass loss observed in TGA. The glass transition was measured to evaluate the effect of the nanoparticles on the polymeric chain’s mobility. The average glass transition temperatures were 54.8, 63.4, and 60.2 °C for the starch films, 1%, and 3% nanocomposite biopolymer films, respectively (Table 2). The films containing 1 and 3% presented an increase in Tg. According to Villalobos et al. [73], a reduction in Tg of an amorphous and/or semicrystalline material indicates an increase in the intermolecular forces between the polymer chains, increasing the local chain flexibility, stimulating the ability of chain rotation, and conferring more strength to the films. Nanocomposites exhibit a higher enthalpy compared to the matrix (Table 2), which could be related to the formation of bonds between the polymeric chains and the reinforcing nanoparticles, hence, more energy is required to break them down. This result is in accordance with the results obtained by DSC and FTIR.

### 3.4. FTIR Analysis of Nanocomposite Biopolymer Films

**Figure 5:**
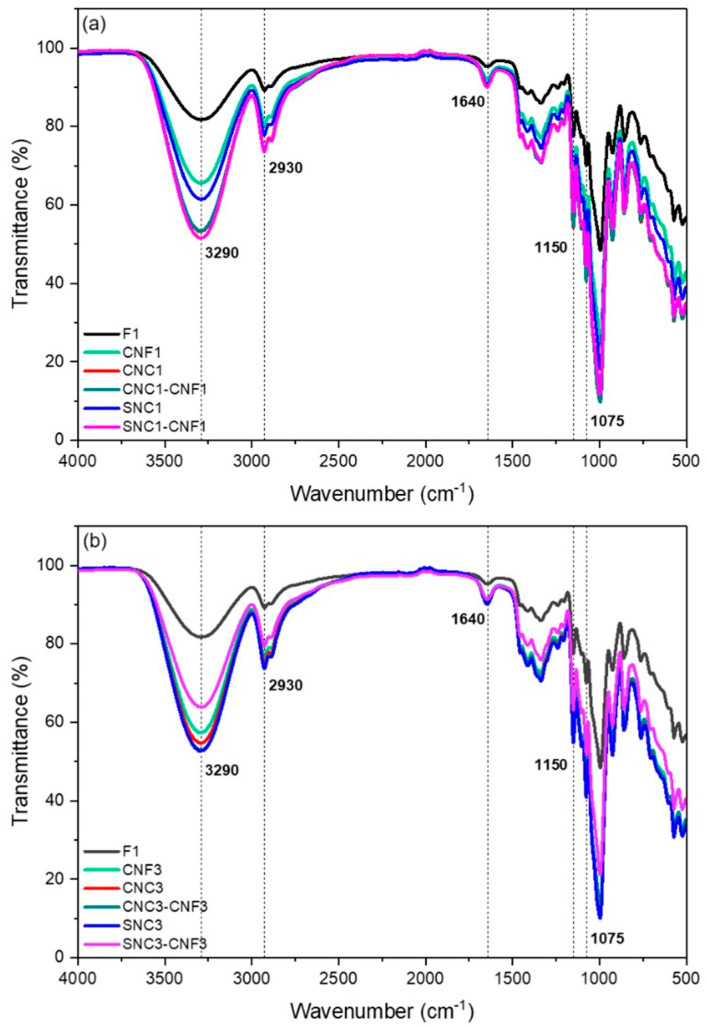
FTIR spectra of the nanocomposite biopolymer films: (**a**) 1% and (**b**) 3%.

The intermolecular interaction among the materials composing the films was analyzed by FTIR, and the results are shown in Figure 5. The nanocomposite biopolymer films containing CNF, CNC, and SNC nanostructures as fillers exhibited almost the same FTIR spectra as the F1 film due to the chemical similarities between starch and cellulose. However, the relative different bandwidth of stretching vibrations for –OH groups in the FTIR spectra of nanocomposite biopolymer films compared to the F1 starch film decreased, indicating that the hydrogen bonding between starch molecules was partially destroyed because of the addition of nanostructures.

A broad and strong adsorption band centered around 3290 cm^−1^ was associated with the stretching vibration of the hydroxyl group (-OH) of the starch films [68,74]. In all films containing nanostructures, a significant decrease in the intensity of this band was observed compared to formulation F1, which was related to the decrease in the amount of hydroxyl groups in starch films due to of the addition of CNF, CNC, and SNC nanostructures [75]. This reduction was more noticeable for those nanocomposite biopolymer films prepared with 3%. The bands in the region between 2930 and 2890 cm^−1^ corresponded to the stretching vibration of the CH_2_ group [76]. Additionally, the intensity at 1640 cm^−1^, related to the H-O-H bonding of water molecules, slightly decreased in the nanocomposite biopolymer films. Other bands identified included 1335 cm^−1^ (C-OH bending vibration), 1150 cm^−1^ (C-O-H stretching vibration), and a stretching vibration band of the C-O group of glycerol, located at 1130 cm^−1^. Signals at 994 and 761 cm^−1^ corresponded to the glycosidic bonds of starch and cellulose [45,75].

Furthermore, the wavenumber of the peak for the C–O stretching vibrations shifted from 994 to 998 in the nanocomposite biopolymer films, suggesting that new interactions between the nanostructures and starch molecules were promoted as a result of the addition of CNFs, CNCs, and SNCs into starch [77]. The interactions between the films and nanostructures (because of their chemical functional groups) were confirmed by the intensity of the bands from the FTIR spectra. As the nanofiber (CNF), cellulose nanocrystal (CNC), and starch nanocrystal (SNC) concentration increased from 1 to 3%, the OH- band increased its intensity, assuming a better interaction between the film starch matrix and the nanostructures. When the concentration of the combined nanostructures (SNC-CNF) increased from 1 to 3% the intensity of the OH- band decreased, probably due to the interaction between the nanostructures themselves compared to the interaction of the nanostructures (SNC-CNF) with the starch film matrix. In this sense, the interactions of the starch film matrix with the nanostructures have an impact on the mechanical properties of starch. Then, as the CNF increased, the tensile strength increased, confirming the better interaction film with the nanostructures, but the elongation at break decreased, meaning that less looseness of the film was present.

### 3.5. Mechanical Properties of the Nanocomposite Biopolymer Films

**Figure 6:**
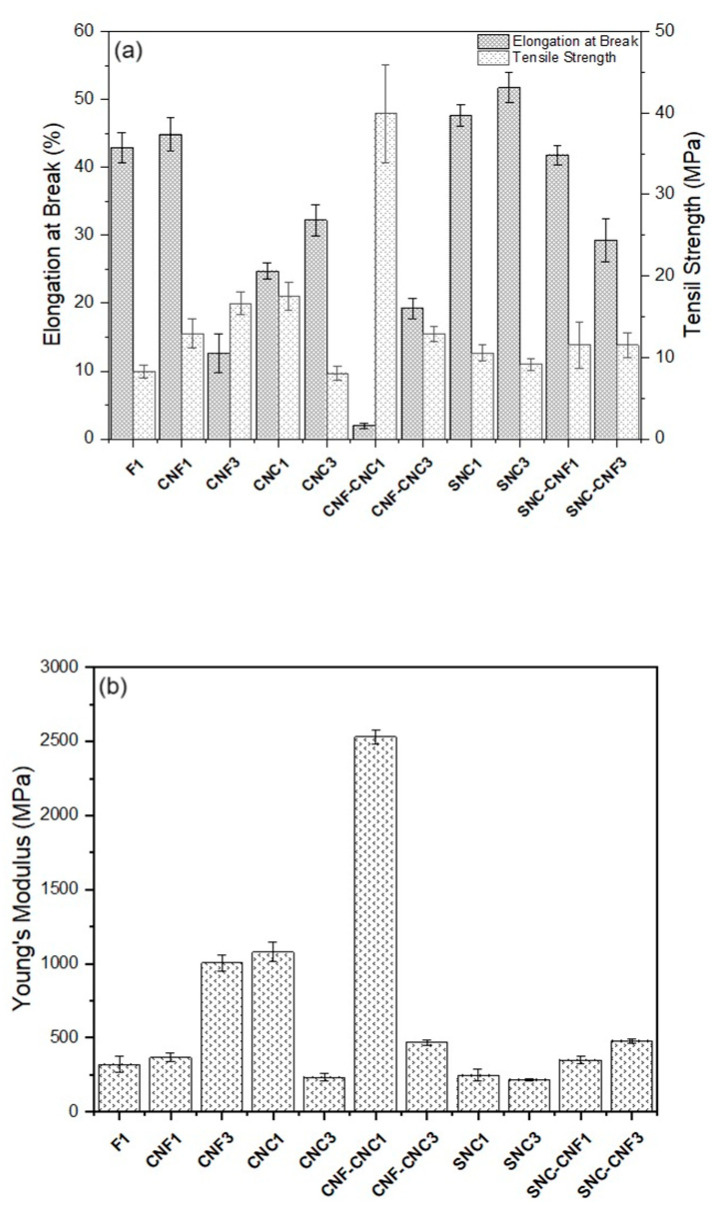
(**a**) Tensile strength, elongation at break, and (**b**) Young’s modulus of the nanocomposite biopolymer films.

**Figure 7:**
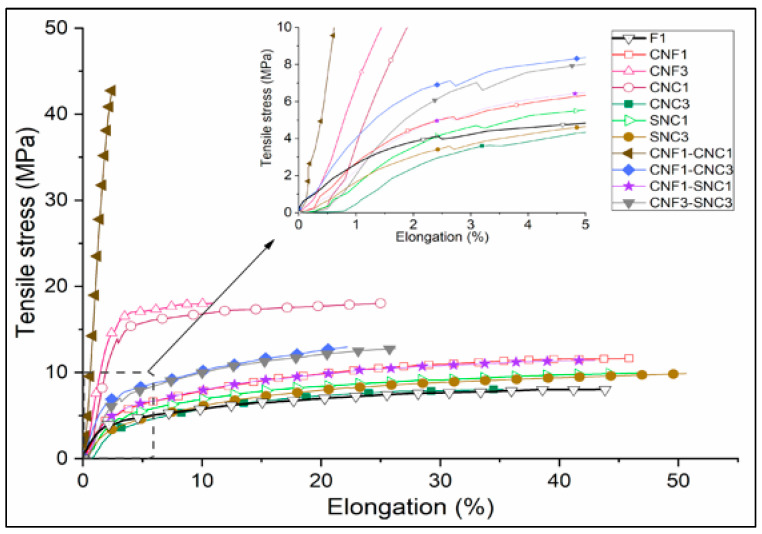
Representative tensile stress versus elongation curves of the nanocomposites.

The effect of type and the content of nanostructures on the mechanical properties of *Commelina coelestis* Willd nanocomposite biopolymer films are shown in Figure 6 and Figure 7. The mean values and standard deviation of the mechanical properties including tensile strength (TS), elongation at break (EB), and Young’s modulus (Y) are reported in Table 3.

Cellulose nanofibers and nanocrystals improved the mechanical properties of the nanocomposite biopolymer films. The reinforcing effect can be analyzed from the increase in Y of the nanocomposites, which is associated with their stiffness. In this sense, the CNF_3_ and CNC_1_ nanocomposites containing one type of particle significantly improved the stiffness of the starch film by tripling this property compared to F1. Regarding the composites with combined particles, CNC_1_-CNF_1_ showed the highest Y value (eight times higher than F1).

The TS of the above samples had the same trend as Y. The enhancement in TS can be explained by the high elastic modulus of the cellulose nanocrystals (CNCs) and the presence of active functional hydroxyl groups on its surface, which interact with the starch matrix to form a rigid network. Furthermore, the better reinforcing capability observed in CNC_1_ compared to CNF_3_ was due to its higher crystallinity and more active functional groups [36].

As expected, compared to F1, the elongation of the CNC_1_-CNF_1_ composite was 95% lower. This sample had such a low elongation that its behavior was fragile and no plastic zone was observed in the stress–elongation curve, opposite to the behavior of all other nanocomposites (Figure 7). CNF_3_ and CNC_1_ had an elongation of 71% and 42% lower than F1, respectively. The interactions of the nanofibers (CNFs) and nanocrystals (CNCs) with the starch films led to a reduction in the mobility of the polymer chains, thus reducing their ductility [78]. The CNF_1_ nanocomposite had a similar elongation to F1 and slightly improved stiffness, but this was not significantly different compared to F1.

The TS values of CNC_3_, SNC_1_, and SNC_3_ were like F1, and Y was similar between SNC_1_ and F1, and lower in CNC_3_ and SNC_3_ with respect to F1 (27% and 32%, respectively). This could be explained by the agglomeration of the nanoparticles at a concentration of 3 wt%, hindering the reinforcement effect [79]. However, this behavior was not observed in the CNF_3_ composite because TS and Y were higher than those of F1. This behavior suggests that the nanofibers were more easily dispersed than the nanocrystals.

The *Commelina coelestis* Willd starch film (F1) had a TS and EB higher than the rice starch film (1.83 ± 0.13 MPa and 5.9 ± 0.1%, respectively) reported by Martins et al. [50]. Unlike our work, starch nanocrystals (SNCs) significantly improved the TS and EB of the native rice starch films. Nevertheless, the addition of SNC was lower (0.1 and 0.3%), and the nanocrystals were of rice and potato. Mukurumbira et al. [80] reported that the amadumbre starch nanocrystals (A-SCNs), in concentrations higher than 2.5%, reduced the TS values of the amadumbre and potato starch films. This was because the low concentration of A-SNCs allowed a uniform distribution of starch nanocrystals to be achieved, while at high concentrations, the A-SNCs aggregated, reducing their active surface area to interact with the matrix. The TS values of the SCNs in our work were similar to those of 8.11 ± 1.67 MPa at 2.5% wt of A-SNCs in the amadumbre starch film reported by Mukurumbira et al. [80].

Comparing the properties of the nanocomposite biopolymer films obtained in this work with synthetic films used in the food packaging industry, it was observed that the elongation at break of the nanocomposites was in the range of the values reported for HDPE (20–50%), while the tensile strength was in the range of LDPE (7–25 MPa) and EVA (6–19 MPa) [81]. However, in the CNF_3_ and notably in CNF_1_-CNC_1_ nanocomposites, the elongation was considerably less, at 12.6 and 1.9%, respectively. In the case of CNF_1_-CNC_1_, the elongation at the Y values was like that of polystyrene (PS), which were 2–3% and 2700–3400 MPa [81]. PS is a material well-known for its high resistance but low ductility, so is therefore widely applicable in thermoformed packaging and is limited to flexible packaging, such as bags obtained by blow-molding processes, so CNF_1_-CNC_1_ could have limited applications where flexibility is required. In the same sense, the CNC_1_ nanocomposite could be the most versatile, because the Y and elongation (1080 MPa, 24.7%) were similar to that of HDPE (980 MPa, 20–50%).

## 4. Conclusions

This study was motivated by the need to find a substitute (biodegradable starch film) for the conventional synthetic plastic currently used in food packaging that is considered to have a short shelf life. Starch from the root of *Commelina coelestis* Willd is a natural polymer with outstanding biocompatible characteristics and can be used as both a matrix and reinforcement material for the development of new nanocomposite biopolymer films.

It was established that the properties of the films are directly related to the concentration and type of nanostructure used.

The hydration, thermal, and mechanical properties were improved with the incorporation of CNF, CNC, and SNC nanostructures. It should be noted that the incorporation of 1% nanostructure presented a better distribution and integrity in the matrix, homogeneity, and low opacity value. The nanocomposite biopolymer films prepared with 1% of the CNF-CNC mixture exhibited the highest tensile strength and Young’s modulus values. The nanocomposites studied exhibited mechanical properties comparable to the main synthetic polymers used in food packaging.

Nanostructures obtained from *Commelina coelestis* Willd are excellent reinforcement options for thermoplastic starch films. These results contribute to improving the performance of films by developing nanocomposite materials for future food packaging applications.

## Figures and Tables

**Table 1:** Swelling capacity, solubility, opacity, and roughness parameters of the nanocomposite biopolymer films.

Film Samples		Property			Roughness Parameters	
	Thickness (mm)	Swelling Capacity (%)	Water Solubility (%)	Opacity (*Abs*_600_ mm^−1^)	*R_a_* (nm)	*R_q_* (nm)
F_1_	0.11 ± 0.01 ^a^	4.2 ± 0.6 ^a^	28.7 ± 1.2 ^a^	2.2 ± 0.2 ^a^	6.87 ± 0.0 ^a^	8.58 ± 0.0 ^a^
CNF_1_	0.09 ± 0.01 ^a^	2.6 ± 0.7 ^b^	23.9 ± 0.7 ^b^	2.5 ± 0.0 ^b^	5.86 ± 0.8 ^b^	7.39 ± 0.5 ^b^
CNF_3_	0.10 ± 0.01 ^a^	1.5 ± 0.1 ^c^	23.3 ± 0.7 ^b^	3.7 ± 0.3 ^c^	9.14 ± 0.0 ^c^	11.39 ± 0.0 ^c^
CNC_1_	0.11 ± 0.01 ^a^	1.5 ± 0.4 ^c^	23.8 ± 0.2 ^b^	2.6 ± 0.1 ^b^	5.22 ± 0.1 ^b^	6.65 ± 0.4 ^b^
CNC_3_	0.10 ± 0.01 ^a^	2.6 ± 0.9 ^b^	24.7 ± 0.3 ^b^	3.4 ± 0.3 ^c^	8.12 ± 0.2 ^d^	10.27 ± 0.0 ^c^
CNC_1_-CNF_1_	0.10 ± 0.02 ^a^	1.0 ± 0.3 ^d^	28.9 ± 1.2 ^a^	2.5 ± 0.0 ^b^	10.38 ± 0.4 ^c^	12.88 ± 0.1 ^d^
CNC_3_-CNF_3_	0.09 ± 0.01 ^a^	2.1 ± 0.1 ^e^	24.2 ± 0.1 ^b^	3.5 ± 0.1 ^c^	8.61 ± 0.1 ^d^	10.67 ± 0.1 ^c^
SNC_1_	0.11 ± 0.01 ^a^	1.7 ± 0.1 ^c^	23.6 ± 0.8 ^b^	2.5 ± 0.1 ^b^	9.22 ± 0.0 ^c^	11.48 ± 0.0 ^c^
SNC_3_	0.09 ± 0.01 ^a^	1.8 ± 0.1 ^c^	31.6 ± 0.9 ^c^	6.2 ± 0.2 ^d^	10.18 ± 0.0 ^c^	12.72 ± 0.0 ^d^
SNC_1_-CNF_1_	0.12 ± 0.01 ^a^	1.9 ± 0.1 ^c^	27.2 ± 0.6 ^a^	2.4 ± 0.0 ^a^	6.29 ± 0.0 ^b^	8.10 ± 0.0 ^a^
SNC_3_-CNF_3_	0.27 ± 0.01 ^b^	2.0 ± 0.2 ^c^	26.9 ± 1.5 ^a^	3.2 ± 0.0 ^c^	7.76 ± 0.0 ^d^	9.58 ± 0.0 ^c^

Values with different letters in the same row indicate a significant difference (*p* ≤ 0.05).

**Table 2:** Thermal analysis of the nanocomposite biopolymer films analyzed by DSC and TGA.

Film Samples	DSC			TGA		
	T_g_ (°C)	T_max_ (°C)	T_End_ (°C)	ΔH (J/g)	T_max_ (°C)	Degradation at T_max_ (%)
F_1_	54.8	196.8	207.0	27.8	303.1	63.5
CNF_1_	69.9	202.6	208.7	30.1	307.15	65.7
CNF_3_	59.0	207.0	212.0	32.4	310.1	78.7
CNC_1_	60.7	207.7	212.4	48.0	308.1	73.6
CNC_3_	60.0	200.6	208.4	64.8	316.1	45.2
CNC_1_-CNF_1_	66.6	202.8	207.9	58.5	305.1	76.5
CNC_3_-CNF_3_	67.4	204.5	209.6	36.8	307.1	76.5
SNC_1_	67.7	206.2	218.2	47.0	310.5	67.3
SNC_3_	227.6	228.3	231.6	32.3	309.3	67.7
SNC_1_-CNF_1_	57.9	201.5	213.1	73.1	305.7	62.1
SNC_3_-CNF_3_	58.0	210.1	217.3	34.7	306.0	68.0

**Table 3:** Mechanical properties of the nanocomposite biopolymer films.

Nanocomposite Biopolymer Films	Tensile Strength (TS)	Elongation at Break (EB)	Young’s Modulus (Y)
	(MPa)	(%)	(MPa)
F1	8.3 ± 0.8 ^a^	42.9 ± 2.3 ^a^	319 ± 55 ^a^
CNF_1_	13.0 ± 1.8 ^b^	44.9 ± 2.5 ^a^	367 ± 29 ^a^
CNF_3_	16.6 ± 1.4 ^b,c^	12.6 ± 2.9 ^b^	1004 ± 52 ^b^
CNC_1_	17.5 ± 1.7 ^c^	24.7 ± 1.2 ^c^	1080 ± 53 ^b^
CNC_3_	8.0 ± 0.9 ^a^	32.2 ± 2.3 ^d^	233 ± 24 ^c^
CNC_1_-CNF_1_	39.9 ± 6.0 ^d^	1.9 ± 0.4 ^f^	2530 ± 45 ^d^
CNC_1_-CNF_3_	12.9 ± 0.9 ^b,c^	19.2 ± 1.5 ^g^	469 ± 20 ^e^
SNC1	10.5 ± 0.9 ^a,b^	47.6 ± 1.5 ^a,e^	249 ± 41 ^a,c^
SNC_3_	9.1 ± 0.7 ^a,b^	51.8 ± 2.8 ^e^	217 ± 6 ^c^
SNC_1_-CNF_1_	11.5 ± 2.9 ^a,b^	41.8 ± 1.4 ^a^	348 ± 25 ^a^
SNC_3_-CNF_3_	11.5 ± 1.5 ^a,b^	29.3 ± 3.2 ^c,d^	479 ± 15 ^e^

Different letters in the same column indicate a statistically significant difference (*p* < 0.05).

## Data Availability

The original contributions presented in the study are included in the article/Supplementary Materials, further inquiries can be directed to the corresponding authors.

## Supplementary Materials

The following supporting information can be downloaded at: https://www.mdpi.com/article/10.3390/foods13244129/s1, Figure S1: X-ray diffractogram of (a) starch, and SNC, (b) cellulose, CNF and CNC nanocrystals.

## References

[1] Luzi F., Fortunati E., Giovanale G., Mazzaglia A., Torre L., Balestra G.M. (2017). Cellulose nanocrystals from *Actinidia deliciosa* pruning residues combined with carvacrol in PVA_CH films with antioxidant/antimicrobial properties for packaging applications. Int. J. Biol. Macromol..

[2] Baniasadi H., Äkräs L., Madani Z., Silvenius F., Fazeli M., Lipponen S., Vapaavuori J., Seppälä J. (2024). Development and characterization of polylactic acid/starch biocomposites—From melt blending to preliminary life cycle assessment. Int. J. Biol. Macromol..

[3] Rojas-Lema S., Nilsson K., Trifol J., Langton M., Gomez-Caturla J., Balart R., Garcia-Garcia D., Moriana R. (2021). Faba bean protein films reinforced with cellulose nanocrystals as edible food packaging material. Food Hydrocoll..

[4] da Rocha M., de Souza M.M., Prentice C. (2018). Biodegradable films: An alternative food packaging. Food Packaging and Preservation.

[5] Kanth S., Puttaiahgowda Y.M. (2022). Current State and Future Perspectives of Starch Derivatives and Their Blends as Antimicrobial Materials. Starch-Starke.

[6] Abera G., Woldeyes B., Demash H.D., Miyake G. (2020). The effect of plasticizers on thermoplastic starch films developed from the indigenous Ethiopian tuber crop Anchote (*Coccinia abyssinica*) starch. Int. J. Biol. Macromol..

[7] Fitch-Vargas P.R., Aguilar-Palazuelos E., Zazueta-Morales J.d.J., Vega-García M.O., Valdez-Morales J.E., Martínez-Bustos F., Jacobo-Valenzuela N. (2016). Physicochemical and Microstructural Characterization of Corn Starch Edible Films Obtained by a Combination of Extrusion Technology and Casting Technique. J. Food Sci..

[8] Nordin N., Othman S.H., Rashid S.A., Basha R.K. (2020). Effects of glycerol and thymol on physical, mechanical, and thermal properties of corn starch films. Food Hydrocoll..

[9] Marichelvam M.K., Jawaid M., Asim M. (2019). Corn and rice starch-based bio-plastics as alternative packaging materials. Fibers.

[10] Thirathumthavorn D., Thongunruan W. (2014). Incorporation of rice starch affecting on morphology, mechanical properties and water vapor permeability of glutelin-based composite films. J. Food Process. Preserv..

[11] Farahnaky A., Saberi B., Majzoobi M. (2013). Effect of glycerol on physical and mechanical properties of wheat starch edible films. J. Texture Stud..

[12] Osés J., Niza S., Ziani K., Maté J.I. (2009). Potato starch edible films to control oxidative rancidity of polyunsaturated lipids: Effects of film composition, thickness and water activity. Int. J. Food Sci. Technol..

[13] Saberi B., Vuong Q.V., Chockchaisawasdee S., Golding J.B., Scarlett C.J., Stathopoulos C.E. (2017). Physical, Barrier, and Antioxidant Properties of Pea Starch-Guar Gum Biocomposite Edible Films by Incorporation of Natural Plant Extracts. Food Bioprocess Technol..

[14] Chandla N.K., Saxena D.C., Singh S. (2017). Amaranth (*Amaranthus* spp.) starch isolation, characterization, and utilization in development of clear edible films. J. Food Process. Preserv..

[15] Pinzon M.I., Garcia O.R., Villa C.C. (2018). The influence of *Aloe vera* gel incorporation on the physicochemical and mechanical properties of banana starch-chitosan edible films. J. Sci. Food Agric..

[16] García-Guzmán L., Velazquez G., Velazquez-Martínez I., Alpizar-Reyes E., Castaño J., Guadarrama-Lezama A.Y. (2024). Thermodynamics of water vapor sorption of fiber-reinforced starch films. Cellulose.

[17] Zavala M., Pérez S., Pérez C., Vargas R., Pérez R. (1998). Antidiarrhoeal activity of *Waltheria americana*, *Commelina coelestis* and *Alternanthera repens*. J. Ethnopharmacol..

[18] Ali A., Xie F., Yu L., Liu H., Meng L., Khalid S., Chen L. (2018). Preparation and characterization of starch-based composite films reinfoced by polysaccharide-based crystals. Compos. Part B Eng..

[19] Slavutsky A.M., Bertuzzi M.A. (2014). Water barrier properties of starch films reinforced with cellulose nanocrystals obtained from sugarcane bagasse. Carbohydr. Polym..

[20] Fazeli M., Simão R.A. (2018). The effect of cellulose nanofibers on the properties of starch biopolymer. Macromol. Symp..

[21] Fazeli M., Florez J.P., Simão R.A. (2019). Improvement in adhesion of cellulose fibers to the thermoplastic starch matrix by plasma treatment modification. Compos. Part B Eng..

[22] Guimarães M., Teixeira F.G., Tonoli G.H.D. (2018). Effect of the nano-fibrillation of bamboo pulp on the thermal, structural, mechanical and physical properties of nanocomposites based on starch/poly(vinyl alcohol) blend. Cellulose.

[23] Lee H., You J., Jin H.-J., Kwak H.W. (2020). Chemical and physical reinforcement behavior of dialdehyde nanocellulose in PVA composite film: A comparison of nanofiber and nanocrystal. Carbohydr. Polym..

[24] Mukurumbira A., Mariano M., Dufresne A., Mellem J.J., Amonsou E.O. (2017). Microstructure, thermal properties and crystallinity of amadumbe starch nanocrystals. Int. J. Biol. Macromol..

[25] de la Concha B.B.S., Agama-Acevedo E., Nuñez-Santiago M.C., Bello-Perez L.A., Garcia H.S., Alvarez-Ramirez J. (2018). Acid hydrolysis of waxy starches with different granule size for nanocrystal production. J. Cereal Sci..

[26] Tibolla H., Czaikoski A., Pelissari F., Menegalli F., Cunha R. (2020). Starch-based nanocomposites with cellulose nanofibers obtained from chemical and mechanical treatments. Int. J. Biol. Macromol..

[27] Haaj S.B., Thielemans W., Magnin A., Boufi S. (2016). Starch nanocrystals and starch nanoparticles from waxy maize as nanoreinforcement: A comparative study. Carbohydr. Polym..

[28] Santana J.S., Costa K.d.C., Rodrigues P.R., Correia P.R.C., Cruz R.S., Druzian J.I. (2019). Morphological, barrier, and mechanical properties of cassava starch films reinforced with cellulose and starch nanoparticles. J. Appl. Polym. Sci..

[29] Tagliapietra B.L., de Melo B.G., Sanches E.A., Plata-Oviedo M., Campelo P.H., Clerici M.T.P.S. (2021). From micro to nanoscale: A critical review on the concept, production, characterization, and application of starch nanostructure. Starch-Starke.

[30] Perea-Flores M.d.J., Martínez-Luna K.L., Núñez-Bretón L.C., Sarria-Guzmán Y., Jiménez-Guzmán J., Alamilla-Beltrán L., Vivar-Vera G., González-Jiménez F.E. (2022). Modification by lipophilic substitution of Mexican *Oxalis tuberosa* starch and its effect on functional and microstructural properties. J. Food Meas. Charact..

[31] García-Guzmán L., Arzate-Vázquez I., Velazquez G., Díaz-Bandera D., García-Eleno M.A., Castaño J., Guadarrama-Lezama A.Y. (2024). Isolation and Characterization of Starch, Cellulose, and Their Nanostructures Obtained from *Commelina coelestis* Willd Root. J. Polym. Environ..

[32] Chavan P., Sinhmar A., Sharma S., Dufresne A., Thory R., Kaur M., Sandhu K.S., Nehra M., Nain V. (2022). Nanocomposite starch films: A new approach for biodegradable packaging materials. Starch-Starke.

[33] Velásquez-Castillo L.E., Leite M.A., Ditchfield C., Sobral P.J.D.A., Moraes I.C.F. (2020). Quinoa starch nanocrystals production by acid hydrolysis: Kinetics and properties. Int. J. Biol. Macromol..

[34] Coelho C.C.d.S., Silva R.B.S., Carvalho C.W.P., Rossi A.L., Teixeira J.A., Freitas-Silva O., Cabral L.M.C. (2020). Cellulose nanocrystals from grape pomace and their use for the development of starch-based nanocomposite films. Int. J. Biol. Macromol..

[35] Castaño J., Guadarrama-Lezama A.Y., Hernández J., Colín-Cruz M., Muñoz M., Castillo S. (2017). Preparation, characterization and antifungal properties of polysaccharide–polysaccharide and polysaccharide–protein films. J. Mater. Sci..

[36] Zhang L., Zhao J., Zhang Y., Li F., Jiao X., Li Q. (2021). The effects of cellulose nanocrystal and cellulose nanofiber on the properties of pumpkin starch-based composite films. Int. J. Biol. Macromol..

[37] Ren L., Yan X., Zhou J., Tong J., Su X. (2017). Influence of chitosan concentration on mechanical and barrier properties of corn starch/chitosan films. Int. J. Biol. Macromol..

[38] Wang T., Zhao Y. (2021). Optimization of bleaching process for cellulose extraction from apple and kale pomace and evaluation of their potentials as film forming materials. Carbohydr. Polym..

[39] Ghanbari A., Tabarsa T., Ashori A., Shakeri A., Mashkour M. (2018). Thermoplastic starch foamed composites reinforced with cellulose nanofibers: Thermal and mechanical properties. Carbohydr. Polym..

[40] Jia R., Cui C., Gao L., Qin Y., Ji N., Dai L., Wang Y., Xiong L., Shi R., Sun Q. (2023). A review of starch swelling behavior: Its mechanism, determination methods, influencing factors, and influence on food quality. Carbohydr. Polym..

[41] Bangar S.P., Ali N.A., Olagunju A.I., Pastor K., Ashogbon A.O., Dash K.K., Lorenzo J.M., Ozogul F. (2023). Starch-based noodles: Current technologies, properties, and challenges. J. Texture Stud..

[42] El Halal S.L.M., Bruni G.P., Evangelho J.A.D., Biduski B., Silva F.T., Dias A.R.G., Zavareze E.d.R., Luvielmo M.d.M. (2018). The properties of potato and cassava starch films combined with cellulose fibers and/or nanoclay. Starch-Starke.

[43] Avérous L., Fringant C., Moro L. (2001). Plasticized starch–cellulose interactions in polysaccharide composites. Polymer.

[44] Yadav M., Chiu F.-C. (2019). Cellulose nanocrystals reinforced κ-carrageenan based UV resistant transparent bionanocomposite films for sustainable packaging applications. Carbohydr. Polym..

[45] Dai L., Zhang J., Cheng F. (2019). Effects of starches from different botanical sources and modification methods on physicochemical properties of starch-based edible films. Int. J. Biol. Macromol..

[46] Yıldırım-Yalçın M., Şeker M., Sadıkoğlu H. (2019). Development and characterization of edible films based on modified corn starch and grape juice. Food Chem..

[47] Casariego A., Souza B., Cerqueira M., Teixeira J., Cruz L., Díaz R., Vicente A. (2009). Chitosan/clay films’ properties as affected by biopolymer and clay micro/nanoparticles’ concentrations. Food Hydrocoll..

[48] Thipchai P., Punyodom W., Jantanasakulwong K., Thanakkasaranee S., Hinmo S., Pratinthong K., Kasi G., Rachtanapun P. (2023). Preparation and characterization of cellulose nanocrystals from bamboos and their application in cassava starch-based film. Polymers.

[49] Piyada K., Waranyou S., Thawien W. (2013). Mechanical, thermal and structural properties of rice starch films reinforced with rice starch nanocrystals. Int. Food Res. J..

[50] Martins P.C., Latorres J.M., Martins V.G. (2022). Impact of starch nanocrystals on the physicochemical, thermal and structural characteristics of starch-based films. LWT.

[51] Balakrishnan P., Gopi S., S S.M., Thomas S. (2018). UV resistant transparent bionanocomposite films based on potato starch/cellulose for sustainable packaging. Starch-Starke.

[52] Gontard N., Duchez C., Cuq J.-L., Guilbert S. (1994). Edible composite films of wheat gluten and lipids: Water vapour permeability and other physical properties. Int. J. Food Sci. Technol..

[53] Li M., Tian X., Jin R., Li D. (2018). Preparation and characterization of nanocomposite films containing starch and cellulose nanofibers. Ind. Crops Prod..

[54] Sogut E., Cakmak H. (2020). Utilization of carrot (*Daucus carota* L.) fiber as a filler for chitosan based films. Food Hydrocoll..

[55] Friesen K., Chang C., Nickerson M. (2015). Incorporation of phenolic compounds, rutin and epicatechin, into soy protein isolate films: Mechanical, barrier and cross-linking properties. Food Chem..

[56] Niu X., Ma Q., Li S., Wang W., Ma Y., Zhao H., Sun J., Wang J. (2021). Preparation and Characterization of Biodegradable Composited Films Based on Potato Starch/Glycerol/Gelatin. J. Food Qual..

[57] Ali A., Yu L., Liu H., Khalid S., Meng L., Chen L. (2017). Preparation and characterization of starch-based composite films reinforced by corn and wheat hulls. J. Appl. Polym. Sci..

[58] Li Z., Wei C. (2020). Morphology, structure, properties and applications of starch ghost: A review. Int. J. Biol. Macromol..

[59] Thiré R.M., Simão R.A., Andrade C.T. (2003). High resolution imaging of the microstructure of maize starch films. Carbohydr. Polym..

[60] Qi K., Cao S., Li C. (2024). Possible interaction between pectin and gluten alters the starch digestibility and texture of wheat bread. Int. J. Biol. Macromol..

[61] Mahardika M., Abral H., Kasim A., Arief S., Hafizulhaq F., Asrofi M., Mahardika M., Abral H., Kasim A., Arief S. (2019). Properties of cellulose nanofiber/bengkoang starch bionanocomposites: Effect of fiber loading. LWT.

[62] Kang M., Tuteja M., Centrone A., Topgaard D., Leal C. (2018). Nanostructured Lipid-Based Films for Substrate-Mediated Applications in Biotechnology. Adv. Funct. Mater..

[63] Gopi S., Amalraj A., Jude S., Thomas S., Guo Q. (2019). Bionanocomposite films based on potato, tapioca starch and chitosan reinforced with cellulose nanofiber isolated from turmeric spent. J. Taiwan Inst. Chem. Eng..

[64] Antoniou J., Liu F., Majeed H., Zhong F. (2015). Characterization of tara gum edible films incorporated with bulk chitosan and chitosan nanoparticles: A comparative study. Food Hydrocoll..

[65] Azeredo H.M., Mattoso L.H.C., Avena-Bustillos R.J., Filho G.C., Munford M.L., Wood D., McHugh T.H. (2010). nanocellulose reinforced chitosan composite films as affected by nanofiller loading and plasticizer content. J. Food Sci..

[66] Ahuja D., Kumar L., Kaushik A. (2021). Thermal stability of starch bionanocomposites films: Exploring the role of esterified cellulose nanofibers isolated from crop residue. Carbohydr. Polym..

[67] Castaño J., Rodríguez-Llamazares S., Carrasco C., Bouza R. (2012). Physical, chemical and mechanical properties of pehuen cellulosic husk and its pehuen-starch based composites. Carbohydr. Polym..

[68] Merino D., Gutiérrez T.J., Alvarez V.A. (2019). Structural and Thermal Properties of Agricultural Mulch Films Based on Native and Oxidized Corn Starch Nanocomposites. Starch-Starke.

[69] Kang S., Xiao Y., Guo X., Huang A., Xu H. (2021). Development of gum arabic-based nanocomposite films reinforced with cellulose nanocrystals for strawberry preservation. Food Chem..

[70] García N.L., Ribba L., Dufresne A., Aranguren M., Goyanes S. (2011). Effect of glycerol on the morphology of nanocomposites made from thermoplastic starch and starch nanocrystals. Carbohydr. Polym..

[71] Liu C., Jiang S., Zhang S., Xi T., Sun Q., Xiong L. (2016). Characterization of edible corn starch nanocomposite films: The effect of self-assembled starch nanoparticles. Starch-Starke.

[72] Fazeli M., Lipponen J. (2022). Developing Self-Assembled Starch Nanoparticles in Starch Nanocomposite Films. ACS Omega.

[73] Villalobos K., Rojas H., González-Paz R., Granados D.B., González-Masís J., Baudrit J.V., Corrales-Ureña Y.R. (2017). Production of starch films using propolis nanoparticles as novel bioplasticizer. J. Renew. Mater..

[74] Sakkara S., Nataraj D., Venkatesh K., Xu Y., Patil J.H., Reddy N. (2020). Effect of pH on the physicochemical properties of starch films. J. Appl. Polym. Sci..

[75] Dai L., Zhang J., Cheng F. (2019). Cross-linked starch-based edible coating reinforced by starch nanocrystals and its preservation effect on graded Huangguan pears. Food Chem..

[76] Meng F., Zhang C., Li J., Wang L. (2021). Self-assembling crystals of an extract of Flos Sophorae Immaturus for improving the antioxidant, mechanical and barrier properties of a cassia gum film. Int. J. Biol. Macromol..

[77] Cao X., Chen Y., Chang P.R., Muir A.D., Falk G. (2008). Starch-based nanocomposites reinforced with flax cellulose nanocrystals. Express Polym. Lett..

[78] Shabanpour B., Kazemi M., Ojagh S.M., Pourashouri P. (2018). Bacterial cellulose nanofibers as reinforce in edible fish myofibrillar protein nanocomposite films. Int. J. Biol. Macromol..

[79] Gamage A., Thiviya P., Mani S., Ponnusamy P.G., Manamperi A., Evon P., Merah O., Madhujith T. (2022). Environmental Properties and Applications of Biodegradable Starch-Based Nanocomposites. Polymers.

[80] Mukurumbira A.R., Mellem J.J., Amonsou E.O. (2017). Effects of amadumbe starch nanocrystals on the physicochemical properties of starch biocomposite films. Carbohydr. Polym..

[81] Bastarrachea L., Dhawan S., Sablani S.S. (2011). Engineering Properties of Polymeric-Based Antimicrobial Films for Food Packaging: A Review. Food Eng. Rev..

