# Supplementary material for: Preparation of Nanocomposite Biopolymer Films from Commelina coelestis Willd Starch and Their Nanostructures as a Potential Replacement for Single-Use Polymers
Source: Foods. 2024 Dec 20;13(24):4129. doi: 10.3390/foods13244129 (PMC11675869; doi:10.3390/foods13244129)
Supplement: Supplementary file 1 [file foods-13-04129-s001.zip › foods-3359061-supplementary.pdf]

Supplementary information

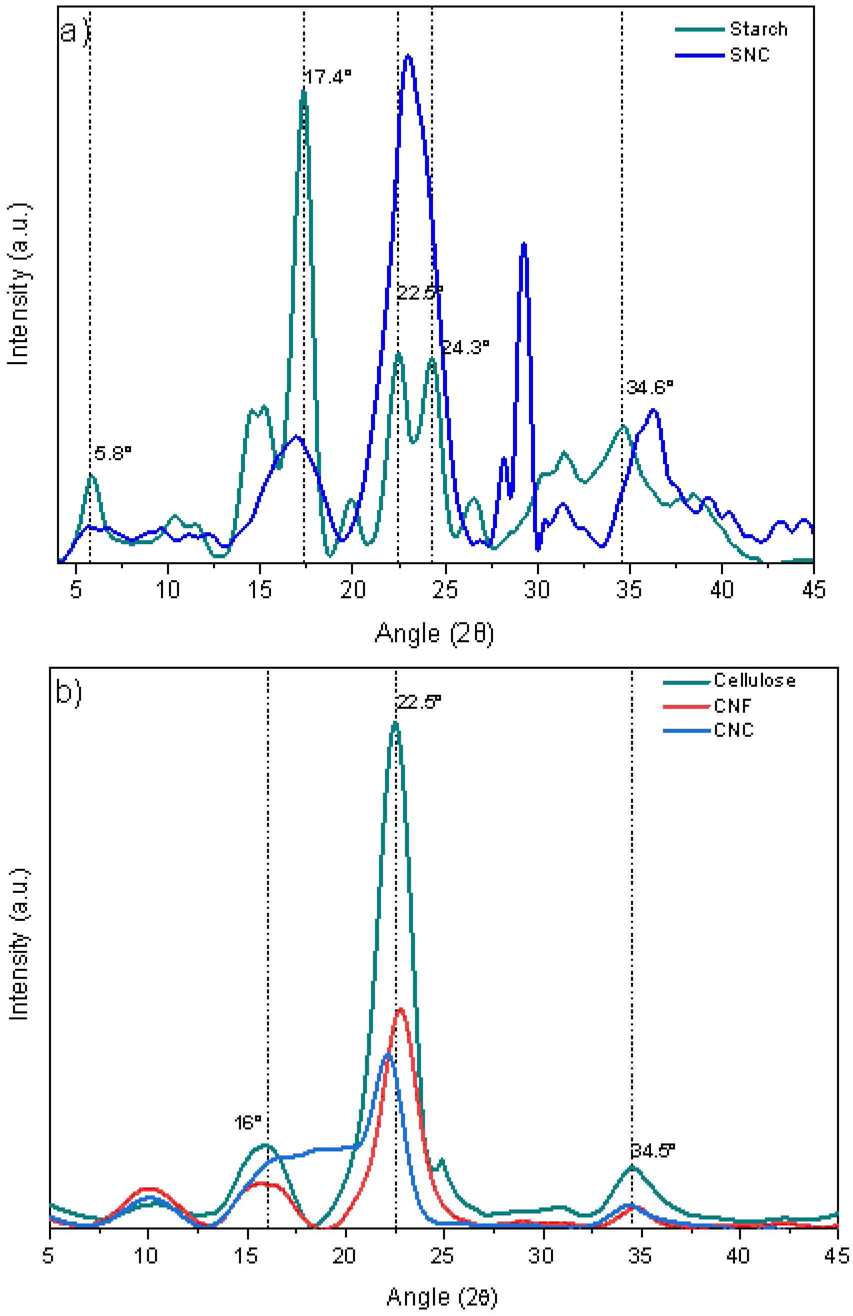

Figure S1. X-ray diffractogram of a) starch, and SNC, b) cellulose, CNF and CNC nanocrystals.
